# Case of Cerebral Abscess

**Published:** 1848

**Authors:** Thos. Hall

**Affiliations:** Toulon, Ind.


### ARTICLE IV.

*Case of Cerebral Abscess.* By THOS. HALL, M.D., Toulon, Ind.

Miss H. J., aged 16, of a tall, slender, and beautiful figure, with black hair, dark brown eyes—of the nervous temperament and strumous diathesis, was attacked suddenly with paralysis, on the evening of the 22d of June. Her maternal ancestors and relatives were healthy; but the brothers and sisters of her father have all died consumptive, at the ages of twenty to thirty-five years. The history of the case, derived from the mother, is, that for six months she has perceived a slight knitting of the eyebrows—with occasional pain on the right side of the head, but usually of very limited duration; her general health was uniformly good until she first complained of the headache; and it was then so slightly disturbed as not to occasion any alarm. She never gave the least evidence of impairment of memory; in fact, the whole of the mental manifestations were unaltered—perfect to the last. She had been afflicted with psoriasis of the left hand, at times, for five years. Her menses were regular, but for a few of the last returns, rather too abundant, and her countenance was anemic.

On the evening mentioned (22d of June) she was at a prayer meeting, and on returning home found she could not raise her left arm—no notice, however, was taken of it until the next morning, when Dr. Smith was called in, and the only manifestation of disease he could detect, was loss of motion of the right arm and leg, (sensation being perfect,) and slight pain in the head, but which was not at that time referred to any particular part---the pulse, tongue, countenance, and skin perfectly natural. He gave her cathartics, and an embrocation to the affected parts;—24th she could slightly move the leg—paralysis of the arm remaining complete—the headache still continued, which is now referred to the right side and top of the head. Constant vomiting, but no epigastric tenderness—several of the common symptoms of hysteria were present. I was now called in to meet Dr. Smith; at once I suspected lesion of the brain, but finding on examination, her appearance, pulse, tongue, pupils, and skin, perfectly natural—her intellect clear, no convulsions, no coma, I was at a loss to determine whether there was organic mischief in the head, or whether it was not altogether one of the many Protean shapes that hysteria delights to assume; on the whole, I leaned to the latter opinion, with which Dr. Smith agreed, and we thought it best to pursue a mild course. The vomiting was arrested by soda draughts; the bowels were kept open by aperients and sinapisms to the spine, &c.

26th. No apparent amendment, nor change in any way, being perceptible under the treatment, and fearing the consequences should we mistake organic disease for hysteria, we concluded to pursue a different course. Her carotids were carefully examined by both, and not the least difference in the beat could be detected—the pupils responded to the light—the pulse natural—the pain in the head referred to the upper and middle portion of the right parietal bone. In consequence of the anemic state of the patient we preferred local to general abstractions of blood and cupped her, first in the back of the neck, then on her right temple, blistered the spine, and gave her mild doses of calomel combined with aperients. Ice was applied over the seat of pain; under this treatment her head was somewhat relieved, and the case looked favorable; but on the night of the 29th we were both called up, and found her to all appearance rapidly sinking—the surface cold—the pulse slow (40 per minute; two beats were quick, and every third beat the heart appeared to labor, contracting very slowly and with great difficulty) and intermitting—she was perfectly sensible—answered every question quickly—her pupils were natural and still responded to the lights—by the free use of stimulants she soon revived; but the pulse still remaining irregular, and being different from any I ever met with before, the opinion was again forced upon me that hysteria, if not the chief, was at least a subordinate cause of the symptoms, and that, if the peculiar character of the heart's action was produced by emotion, a full dose of morphine would bring it back to its normal condition; with Dr. S.'s consent it was given, and we left her till morning, when we found her much better—pulse natural—no pain in the head but the paralysis still remained. Her appetite now returned, and she took food freely, resumed her usual cheerfulness, laughed heartily—her countenance perfectly natural, and under a course of mild tonics she appeared to be rapidly convalescing until the morning of the second of July, when her skin was somewhat dry, and her pulse slightly intermitting—the tonics were suspended—she still was cheerful and took some food. As the day advanced, the pain in the head returned; but she chatted cheerfully with her friends until about five o'clock, P. M., when she became suddenly unconscious, and was dead in a few minutes.

Such is a short but faithful outline of the main features of the case; and, before passing to the post-mortem appearances, I would advise the reader to pause, and, if possible from the description here given, form an opinion of the pathology of it. It is a practice I pursue in reading reported cases, and it has many advantages which I will not stay to mention.

*Post-Mortem Appearances.*—On removing the integuments of the head, we found slight injections of the blood vessels over the upper, middle, and posterior portion of the right parietal bone—when the skull cap was taken off, a corresponding injection was visible on the coverings of the brain; but the surface of the brain itself appeared perfectly healthy. On cutting into the cineritious portion of the brain, we found it natural; in a careful examination of the most superficial portion of the medullary matter, the right side was evidently less injected than the left, and firmer; and on making a deeper incision, we found an encysted abscess containing from two and a half to three ounces of pus, which had burst into the right ventricle; a small ulcer was visible on the upper surface of the cyst; no other morbid appearance, except a slight serous effusion under the pituitary gland.

To my mind there are several interesting features in the case; some of a general character and others personal to Dr. Smith and myself. In the first place—could we by any known method of examination have ascertained the exact condition of the disease by its manifestations during any part of its continuance? If so, we failed to do it. There is no lesion of the brain or spinal column productive of paralysis, but what was thought of and closely investigated, and it differed from every case we had seen and every case on record within our reach; it differs as widely from every case of encysted abscess in the brain recorded by Abercrombie, as they differ from each other. If paralysis marks the commencement of suppuration, which is evidently his opinion, where were the common symptoms characteristic of the inflammatory stage? they were all absent if we except the headache, and that was far from being constant (the knitting of the eyebrows was not seen by either of us during our attendance), unless, as supposed by some, simple inflammation of the brain or its membranes may produce paralysis. What was the precise condition of the brain when Dr. Smith first saw the patient, or, in other words, what occasioned the paralysis? This question I would like to see answered; I cannot satisfy my own mind on the point, although I have thought much and read more in order satisfactorily to solve the difficulty. Another perplexing question, at least to me, is, what caused that sinking condition of the system and the singular pulse on the night of the 29th? I still think they were due to hysteria; although I have thought that if the paralysis was produced by inflammation of the brain, that condition might mark the commencement of suppuration. It is not the least remarkable feature of the case that the mental manifestations were never altered or suspended for one minute from the commencement to the termination of it; no irritability of temper; no impairment of memory; no convulsions, and no coma. In most of these, I believe it differs from every case on record, within my knowledge, where an encysted abscess has been found in the brain after death. The sudden dissolution was probably caused by the cyst giving way and pouring its contents into the ventricle.

